# Associations between the dietary inflammatory index with obesity and body fat in male adolescents

**DOI:** 10.1186/s12902-022-01001-x

**Published:** 2022-05-02

**Authors:** Maryam Gholamalizadeh, Mina Ahmadzadeh, Fatemeh BourBour, Farhad Vahid, Marjan Ajami, Nazanin Majidi, Azadeh Hajipour, Saeid Doaei, Naser Kalantari, Atiyeh Alizadeh, Alireza Mosavi Jarrahi

**Affiliations:** 1 Students Research Committee, Cancer Research Center, Shahid Beheshti University of Medical Sciences, Tehran, Iran; 2 Department of Clinical Nutrition and Dietetics, Faculty of Nutrition and Food Technology, National Nutrition and Food Technology Research Institute, Shahid Beheshti University of Medical Sciences, Tehran, Iran; 3 Population Health Department, Nutrition and Health Research Group, Luxembourg Institute of Health, Strassen, Luxembourg; 4 Department of Food and Nutrition Policy and Planning, National Nutrition and Food Technology Research Institute, School of Nutrition Sciences and Food Technology, Shahid Beheshti University of Medical Sciences, Tehran, Iran; 5 Department of Nutrition, Science and Research Branch, Islamic Azad University, Tehran, Iran; 6 School of Health, Qazvin University of Medical Sciences, Qazvin, Iran; 7 Research Center of Health and Environment, School of Health, Guilan University of Medical Sciences, Rasht, Iran; 8 Department of Community Nutrition, School of Nutrition and Food Sciences, Shahid Beheshti University of Medical Sciences, Tehran, Iran; 9 Department of Pharmacognosy, Faculty of Pharmacy, Tehran University of Medical Sciences, Tehran, Iran; 10 School of Medicine, Shahid Beheshti University of Medical Sciences, Tehran, Iran

**Keywords:** Obesity, Dietary inflammatory index, Body fat, Adolescence, Body composition

## Abstract

**Background:**

Obesity and body composition may be affected by the pro-inflammatory and anti-inflammatory components of diets. The aim of this study was to investigate associations between the dietary inflammatory index (DII) and body fat percentage (BF%) in male adolescents.

**Methods:**

This cross-sectional study was carried out on 535 adolescent boys in Tehran, Iran. Bio-impedance analyzer (BIA) scale was used to measure body mass index (BMI) and body composition. A validated semi-quantitative food frequency questionnaire (FFQ) was used to measure DII.

**Results:**

Participants with higher BF% (≥ 19.2%) had higher BMI (*P* < 0.001), DII, and intake of saturated fatty acids (SFAs), compared with the participants with lower BF%. Participants with a lower DII had significantly higher intakes of fibers (*P* < 0.001) and lower intakes of fats, SFAs, monounsaturated fatty acids (MUFAs), polyunsaturated fatty acids (PUFAs), oleic acid and linoleic acid (*P* < 0.05) compared with the participants with higher DII (*P* < 0.01). High BF% was positively associated to DII (OR = 1.6, CI 95%: 1.1–2.3, *P* = 0.019). Adjustments for age, BMI, physical activity, mothers’ marital status and educational level and the number of siblings did not change the results.

**Conclusions:**

Following a diet with a low inflammatory index score was associated to lower BF% in male adolescents. Thus, a diet rich in anti-inflammatory factors may effectively improve body composition and prevent obesity in adolescents. Further comprehensive studies are necessary to verify these findings and to identify the underlying mechanisms.

## Introduction

Obesity is a multifactorial disorder characterized by excess body fat and a high body mass index (BMI) [1]. More than 18% of children and adolescents aged 5–19 years are overweight or obese worldwide, compared to 4% of this age group in 1975, which represents rapid increases in the prevalence of obesity [2]. A meta-analysis on Iranian adolescents has reported the prevalence of obesity and overweight as 5.5 and 15.1%, respectively [3]. Improved physical activities and appropriate diets as modifiable risk factors are crucial parts of weight management interventions [4]. Dietary intake plays important roles in obesity and its complications such as insulin resistance, hypertension, dyslipidemia, non-alcoholic fatty liver, heart disease, and stroke [5, 6]. A possible mechanism of the associations between obesity and chronic diseases relies on the theory of inflammation and inflammatory markers such as C-reactive protein (CRP), tumor necrosis factor α (TNF-α), and interleukin 6 (IL-6) [7]. For example, studies have reported that excess adipose tissues in obese individuals can increase leptin and cytokines production and decrease anti-inflammatory immune cells, which lead to inflammation [8, 9].

Dietary inflammatory index (DII) has been described as the assessment of the inflammatory potential of the diet [10]. This nutritional tool assessed intakes of 45 pro-inflammatory and anti-inflammatory dietary components [11, 12]. If the DII score is negative, diet is assumed to include anti-inflammatory effects and if this score is positive, diet is suggested to include inflammatory effects [13]. Studies have reported associations between the DII score and obesity in adults [14]. Oliveira et al. have shown that participants in the highest DII quartile are more likely to consume fats, red meats, processed foods, sugars, sweets, and calories and have a higher prevalence of obesity and overweight [14, 15]. Furthermore, DII is positively linked to adipose tissues and is negatively linked to lean tissues in Iranian women [16]. Another study has demonstrated that higher scores of DII are associated with a higher risk of obesity and overweight in childhood [17]. Ruiz-Canela et al. have shown direct associations between the DII and obesity indices, including BMI and waist circumference, and reported that diet may include roles in the development of obesity through inflammatory modulation mechanisms [5]. Since body composition is reported as a better indicator for obesity and weight assessment in adolescents [18] and a few studies have already assessed associations between the DII and body composition [17, 19], the major aim of the current study was to investigate associations between the DII and body fat percentage (BF%) in male adolescents. Diets with higher DII scores were hypnotized to increase BF%.

## Material and methods

Totally, 535 male students aged 12–16 years participated in this cross-sectional study, from August to December 2018. Inclusion criteria included a willingness to participate and written consent of the adolescents and their parents to participate in the study. Adolescents were excluded from the study if they had diseases affecting their diet, weight, and body composition, including diabetes, hyperlipidemia, fatty liver, and cancer. Data on the sociodemographic characteristics of the participants were collected using general questionnaires. Physical Activity (PA) Trackers Mi Band v.2 (MB; Xiaomi, Beijing, China) were used to measure the distance traveled by the participants during the day. These bracelets included three-axis accelerometers that recorded participants’ activities in three axes (horizontal, vertical, and diagonal) in meters [20].

### Body composition and anthropometric measurements

Weight was measured with light clothing using the Seca scale (Seca, Germany) with an accuracy of 100 g. Height was measured with no shoes using a tape meter attached to the wall with a precision of 0.5 cm. The BMI was calculated by dividing weight (kg) by the square of height (m). Levels of fat mass, muscle mass and basal metabolic rate (BMR) were assessed using Omron Body Composition Analyzer (Omron Model BF-511, Omron, Japan). Prerequisites for using BIA were explained to the participants, including the proper posture of the body and limbs (lying on the back, arms abducted at least 30 degrees, legs abducted at nearly 45 degrees), no consumption of alcoholic beverages for at least 12 h, fasting for at least 2 h and avoidance of physical activities for at least 12 h before measurements as well as no intake of drugs affecting body fluid and electrolyte balances [21].

### Dietary intake and dietary inflammatory index (DII) assessments

A validated 168-item semi-quantitative food frequency (FFQ) questionnaire was used to assess the dietary intake of the participants [22]. This questionnaire was completed for each participant through a face-to-face interview by a trained nutritionist. Data collected from FFQ were converted to grams using household measures and then transferred to Nutritionist IV Software for analysis. In addition, daily calorie intakes were assessed using cross-check method of FFQ and 24-h diet recalls for three randomly selected days. To estimate the inflammatory effects of the diets, world standard mean values for the intake of food items were subtracted from the actual intake values for each food item and then divided by the world standard deviation to create a z-score. This value was multiplied by the inflammatory effect score for each food parameter. Scores were summarized for all the food parameters to achieve the overall DII scores. More positive scores showed more pro-inflammatory effects and more negative scores showed more anti-inflammatory effects of the diets. In this study, the following food items were included in the calculation of DII [23]: energy, carbohydrates, proteins, total fat, saturated fatty acids (SFAs), monounsaturated fatty acids (MUFAs), polyunsaturated fatty acids (PUFAs), cholesterol, fibers, oleic acid, linoleic acid, eicosapentaenoic acid (EPA), docosahexaenoic acid (DHA), sodium, potassium, vitamin A, beta-carotene, lutein, lycopene, vitamin C, calcium, iron, vitamin D, vitamin E, vitamin B_12_, thiamin, riboflavin, niacin, vitamin B_6_, folate, biotin, pantothenic acid, vitamin K, magnesium, zinc, and selenium.

### Statistical analyses

Chi-square test and independent t-test were used to analyze categorical and quantitative variables, respectively. To focus on body fat as an outcome, DII was analyzed as both dichotomous (categorized based on the median value of the DII) and continuous variables. Odds ratios (ORs) and 95% confidence intervals (CIs) were estimated for body fat as the outcome and normal and energy-adjusted forms of DII as the independent variable using logistic regression models. A stepwise (forward) selection procedure was used for modeling and variables were selected based on significance or information criteria and background knowledge. Potential confounders were adjusted using the following models of crude (Model A), adjustments for age and BMI (Model B), and additional adjustments for physical activity, mothers’ marital status and educational level, and the number of siblings (Model C). Statistical analysis was carried out using SPSS Software v.21 (IBM, Chicago, USA) and *P* < 0.05 was considered statistically significant.

## Results

General characteristics and levels of macronutrient intakes for the participants with high and low DII are presented in Table 1. Participants with a higher DII (DII ≥ 0.4) had higher metabolic rates (1697.7 ± 237.5 against 1624.7 ± 212.8) and BF% (21.4 ± 10.2 against 8.7 ± 7.7) and lower muscle masses (37.4 ± 4.2 against 38.4 ± 3.5), compared with the participants with a lower DII (DII < 0.4) (*P* < 0.05). Regarding dietary intakes, participants with a lower DII had significantly higher intakes of fibers (21.3 ± 11.8 against 19 ± 6, *P* < 0.001) and lower intakes of dietary fats (97.0 ± 46.7 against 111.5 ± 36.2), SFA (25.5 ± 14.6 against 28.7 ± 9.3), MUFA (36.5 ± 18.9 against 42.5 ± 15.1), PUFA (25.4 ± 15.2 against 30.3 ± 12.4), oleic acid (32.5 ± 18.1 against 39.0 ± 15.0) and linoleic acid (22.9 ± 14.7 against 27.7 ± 12.1), compared with the participants with a higher DII (*P* < 0.05). No significant differences were detected in age, weight, height, BMI, physical activity, and intakes of energy, proteins, carbohydrates, cholesterol, linoleic acid, EPA, DHA, sodium, and potassium of the participants with various DII values. In terms of micronutrients intakes, participants with a lower DII had significantly higher intakes of beta-carotene (3004 ± 3037 against 2441 ± 1447) and lutein (1186 ± 1403 against 956.5 ± 460.1) (*P* < 0.001) (Table 2). Dietary macronutrient intakes of the participants based on the categories of body fat are provided in Table 3. Participants with BF% ≥ 19.2 had higher BMI (29.2 ± 3.3 against 28.8 ± 2.8) (*P* < 0.001), energy-adjusted DII (0.52 ± 0.89 against 0.30 ± 1.1, *P* = 0.013) and intake of SFA (28.8 ± 11.3 against 26.8 ± 9.3 *P* = 0.033), compared with the participants with BF% ˂ 19.2. No significant differences were seen in micronutrient intakes of the groups with various body fat values (Table 4).

**Table 1:** General characteristics and distribution of the macronutrient dietary intakes based on the categories of density DII^*^ (*n* = 535)

	Density DII Categories^**^		**Total**	*P*-value^***^
	**DII > 0.4**	**DII ≥ 0.4**		
Age (y)	14.1 ± 1.3	13.9 ± 0.9	14.1 ± 1.2	0.188
Weight (kg)	79.6 ± 35.5	77.0 ± 10.0	70.1 ± 32.2	0.463
Height (cm)	165.0 ± 4.1	165.6 ± 7.0	165.1 ± 4.8	0.184
BMI (kg/m^2^)	28.6 ± 2.4	28.0 ± 2.9	28.5 ± 2.5	0.050
MR (kcal/d)	1624.7 ± 212.8	1697.7 ± 237	1638.9 ± 219	0.002
Body muscle (%)	38.4 ± 3.5	37.4 ± 4.2	38.2 ± 3.6	0.013
Body fat (%)	18.7 ± 7.7	21.4 ± 10.2	19.2 ± 8.3	< 0.001
Physical activity (m/d)	920.2 ± 621.9	1224.0 ± 2871	979.3 ± 1384	0.05
Number of siblings	2.1 ± 0.5	2.2 ± 0.7	2.1 ± 0.6	0.170
Energy intake (kcal/d)	2463 ± 581.5	2424 ± 816.2	2436 ± 635.5	0.045
Parents’ education (n %)				< 0.001
Illiterate	19 (4.4%)	13 (12.5%)	32 (5.9%)	
Up to diploma	379 (87.9%)	77 (74.0%)	456 (85.2%)	
College education	33 (7.6%)	14 (13.4%)	47 (8.8%)	
Parents marital status (*n* %)				0.301
Widow	1 (0.2%)	1 (0.9%)	2 (0.3%)	
Married	429 (99.6%)	102 (98.2%)	531 (99.4%)	
Divorced	1 (0.2%)	1 (0.9%)	2 (0.3%)	
Protein (g/d)	89.3 ± 22.6	91.5 ± 41.0	89.7 ± 27.1	0.456
Carbohydrate (g/d)	284.0 ± 68.0	280.4 ± 99.1	283.3 ± 75.0	0.665
Total fiber (g/d)	19.0 ± 6.0	21.3 ± 11.8	19.4 ± 7.5	< 0.001
- Soluble fiber (g/d)	0.4 ± 0.2	0.4 ± 0.4	0.4 ± 0.3	0.903
Total fat intake (g/d)	111.5 ± 36.2	97.0 ± 46.7	108.7 ± 38.8	< 0.001
Cholesterol (mg/d)	306.4 ± 156.6	300.4 ± 278.0	305.2 ± 186.2	0.768
SFA (g/d)	28.7 ± 9.3	25.5 ± 14.6	28.1 ± 10.6	< 0.001
MUFA (g/d)	42.5 ± 15.1	36.5 ± 18.9	41.4 ± 16.1	< 0.001
PUFA (g/d)	30.3 ± 12.4	25.4 ± 15.2	29.3 ± 13.1	< 0.001
Oleic acid (g/d)	39.0 ± 15.0	32.5 ± 18.1	37.7 ± 15.8	< 0.001
Linoleic acid (mg/d)	27.7 ± 12.1	22.9 ± 14.7	26.8 ± 12.8	< 0.001
Linolenic acid (mg/d)	0.9 ± 0.7	0.7 ± 0.8	0.9 ± 0.7	0.031
EPA (g/d)	0.01 ± 0.07	0.01 ± 0.06	0.01 ± 0.07	0.453
DHA (g/d)	0.06 ± 0.23	0.04 ± 0.20	0.05 ± 0.22	0.421
Sodium (mg/d)	2735 ± 4755	2499 ± 1377	2689 ± 4310	0.617
Potassium (mg/d)	2590 ± 675.4	2657 ± 1218	2603 ± 808.9	0.446

^*^DII, adjusted for energy

^**^Categorized based on the median

^***^Independent sample t-test was used for comparison f Chi-square test was used for comparison

*BMI* Body mass index, *MR* Metabolic rate, *SFA* Saturated fatty acid, *MUFA* Mono unsaturated fatty acid, *PUFA* Poly unsaturated fatty acid, *EPA* Eicosapentaenoic acid, *DHA* Docosahexaenoic acid

**Table 2:** Distribution of micronutrient dietary intakes based on the categories of density DII^*^ (*n* = 535)

	Density DII category^**^		**Total**	*P*-value ^***^
	**Mean ± SD**		**Mean ± SD**	
	**DII > 0.4**	**DII ≥ 0.4**		
Vitamin A (RAE/d)	922.4 ± 2540	787.3 ± 1763	813.6 ± 1937	0.524
Beta-carotene (mg/d)	3004 ± 3037	2441 ± 1447	2550 ± 1875	< 0.001
Lutein (mg/d)	1186 ± 1403	956.5 ± 460.1	1001 ± 747.3	< 0.001
Lycopene (mg/d)	4499 ± 5796	4609 ± 4996	4588 ± 5156	0.845
Vitamin C (mg/d)	80.0 ± 74.8	72.3 ± 43.6	73.8 ± 51.2	0.176
Calcium (mg/d)	783.5 ± 383.4	784.1 ± 239.9	783.9 ± 273.3	0.986
Iron (mg/d)	16.9 ± 6.7	16.2 ± 3.8	16.3 ± 4.6	0.154
Vitamin D (µg/d)	1.2 ± 1.8	1.3 ± 0.9	1.3 ± 1.9	0.249
Vitamin E (mg/d)	31.7 ± 22.2	36.2 ± 16.9	35.3 ± 18.1	0.022
Thiamin (mg/d)	1.8 ± 0.6	1.7 ± 0.4	1.7 ± 0.5	0.634
Riboflavin (mg/d)	1.9 ± 1.5	1.8 ± 0.6	1.8 ± 0.8	0.211
Niacin (mg/d)	27.0 ± 14.3	26.2 ± 8.4	26.4 ± 9.8	0.441
Vitamin B_6_ (mg/d)	1.8 ± 0.9	1.7 ± 0.4	1.8 ± 0.5	0.228
Folate (µg/d)	465.8 ± 184.8	475.4 ± 114.9	473.5 ± 131.3	0.503
Vitamin B_12_ (µg/d)	9.3 ± 25.9	8.3 ± 18.0	8.5 ± 19.8	0.663
Biotin (mg/day)	24.4 ± 16.5	25.7 ± 10.2	25.5 ± 11.7	0.299
Pantothenic acid (mg/d)	5.0 ± 2.5	5.2 ± 1.5	5.2 ± 1.7	0.264
Vitamin K (µg/d)	166.7 ± 271.1	143.3 ± 116.3	147.8 ± 158.6	0.176
Magnesium (mg/d)	334.2 ± 146.4	316.9 ± 78.4	320.2 ± 95.6	0.098
Zinc (mg/d)	13.9 ± 7.4	13.5 ± 4.2	13.6 ± 5.0	0.469
Selenium (µg/d)	124.2 ± 77.8	122.4 ± 44.7	122.7 ± 52.7	0.752

^*^DII, adjusted for energy

^**^Categorized based on the median

^***^Independent sample t-test was used for mean comparison

*DII* Dietary inflammatory index, *RAE* Retinol activity equivalents

**Table 3:** General characteristics and distribution of macronutrient and electrolyte dietary intakes based on the categories of body fat (*n* = 535)

	Body fat category^*^		*P*-value^**^
	**Mean ± SD**		
	**Body fat > 19.2**	**Body fat ≥ 19.2**	
Age (y)	14.1 ± 1.5	14.0 ± 1.0	0.135
Weight (kg)	78.6 ± 9.1	79.4 ± 40.7	0.783
Height (cm)	164.9 ± 5.4	165.2 ± 4.4	0.573
BMI (kg/m^2^)	28.8 ± 2.8	29.2 ± 3.3	< 0.001
Normal DII^***^	0.05 ± 1.26	0.05 ± 0.96	0.993
Density DII^****^	0.30 ± 1.1	0.52 ± 0.89	0.013
Total energy intake (kcal/d)	2386 ± 660.2	2468 ± 617.8	0.145
Total protein intake (g/d)	89.6 ± 31.8	89.8 ± 23.7	0.954
Total carbohydrate intake (g/d)	276.7 ± 84.9	287.5 ± 67.7	0.103
Total fiber (g/d)	19.5 ± 8.9	19.3 ± 6.5	0.758
Soluble fiber (g/d)	0.38 ± 0.31	0.41 ± 0.30	0.224
Total fat intake (g/d)	106.1 ± 37.8	110.4 ± 39.5	0.216
Cholesterol intake (mg/d)	313.9 ± 233.3	299.6 ± 148.1	0.385
SFA (g/d)	26.8 ± 9.3	28.8 ± 11.3	0.033
MUFA (g/d)	40.4 ± 15.5	42.0 ± 16.4	0.256
PUFA (g/d)	29.0 ± 13.6	29.6 ± 12.8	0.610
Oleic acid (mg/d)	36.7 ± 15.4	38.4 ± 16.1	0.233
Linoleic acid (mg/d)	26.4 ± 13.1	27.1 ± 12.6	0.542
Linolenic acid (mg/d)	0.91 ± 0.94	0.90 ± 0.56	0.865
EPA (mg/d)	0.01 ± 0.08	0.01 ± 0.06	0.852
DHA (mg/d)	0.05 ± 0.27	0.06 ± 0.19	0.765
Sodium (mg/d)	2468 ± 1082	2831 ± 5460	0.342
Potassium (mg/d)	2586 ± 923.8	2641 ± 726.4	0.695

^*^Categorized based on the median

^**^Independent sample t-test was used for mean comparison

^*^Normal DII, not adjusted DII

^**^Density DII, DII adjusted for energy

*DII* Dietary inflammatory index, *BMI* Body mass index, *SFA* Saturated fatty acid, *MUFA* Monounsaturated fatty acid, *PUFA* Polyunsaturated fatty acid, *EPA* Eicosapentaenoic acid, *DHA* Docosahexaenoic acid

**Table 4:** Distribution of the micronutrient dietary intakes based on the categories of body fat (*n* = 535)

	Body fat category^*^		*P*-value^**^
	**Mean ± SD**		
	**Body fat > 19.2**	**Body fat ≥ 19.2**	
Vitamin A (RAE/day)	924.0 ± 2770	752.6 ± 1126	0.322
Beta-carotene (mg/d)	2590 ± 1937	2525 ± 1811	0.693
Lutein (mg/d)	1007 ± 688.2	996.8 ± 784.2	0.868
Lycopene (mg/d)	4278 ± 5836	4788 ± 4663	0.265
Vitamin C (mg/d)	78.4 ± 68.4	70.8 ± 35.7	0.095
Calcium (mg/d)	765.2 ± 269.4	796.1 ± 275.5	0.202
Iron (mg/d)	16.5 ± 5.7	16.2 ± 3.6	0.573
Vitamin D (µg/d)	1.2 ± 1.2	1.3 ± 1.2	0.391
Vitamin E (mg/d)	34.7 ± 18.2	35.7 ± 18.1	0.513
Thiamin (mg/d)	1.7 ± 0.5	1.7 ± 0.4	0.973
Riboflavin (mg/d)	1.8 ± 0.9	1.8 ± 0.7	0.606
Niacin (mg/d)	26.3 ± 11.6	26.3 ± 8.5	0.974
Vitamin B6 (mg/d)	1.8 ± 0.6	1.7 ± 0.5	0.816
Folate (µg/d)	466.3 ± 141.5	478.2 ± 124.2	0.308
Vitamin B12 (µg/d)	9.5 ± 28.4	7.9 ± 11.2	0.365
Biotin (mg/d)	25.4 ± 13.5	25.5 ± 10.3	0.951
Pantothenic acid (mg/d)	5.1 ± 2.2	5.2 ± 1.4	0.757
Vitamin K (µg/d)	148.1 ± 161.6	147.7 ± 156.8	0.978
Magnesium (mg/d)	320.5 ± 113.6	320.1 ± 82.0	0.966
Zinc (mg/d)	13.7 ± 6.1	13.5 ± 4.1	0.674
Selenium (µg/d)	122.9 ± 64.4	122.6 ± 43.7	0.948

^*^Categorized based on the median

^**^ Independent sample t-test was used for mean comparison

*DII* Dietary inflammatory index, *RAE* Retinol activity equivalents

Associations between the DII (normal and energy-adjusted) and body fat based on the continuous variables and cut-off points of DII are presented in Table 5. Regarding associations between the body fat and DII categories, groups with normal DII ≥ -0.12 and density DII ≥ 0.4 were at 1.6 and 2.5 times higher odds of high body fats, compared with the groups with DII < -0.12 (OR = 1.6, CI 95% 1.1–2.3, *P* = 0.019) and DII < 0.4 (OR = 2.5, CI 95% 1.6–3.9, *P* < 0.001), respectively. Results were still significant after adjustments for age and BMI. Further adjustments for the mother’s marital status and educational level, and the number of children (siblings) did not change the results. Results from modeling normal DII as a continuous variable showed that the participants with a higher DII had nonsignificant positive higher body fat values, compared with the participants with a lower DII. Results of the density DII as a continuous variable demonstrated that participants with a higher DII had higher body fat values, compared with the participants with a lower DII (OR = 1.2, CI 95% 1.1–1.4, *P* = 0.015). Results were still significant after adjusting for age and BMI (OR = 1.2, CI 95% 1.1–1.5, *P* = 0.021). Further adjustments for the mother's marital status and educational level, and the number of siblings did not change the results (OR = 1.2, CI 95% 1.1–1.5, *P* = 0.033).

**Table 5:** The ORs and 95% CIs for the associations between the DII (normal and energy-adjusted) and body fat (*n* = 535)

	Normal DII category (OR, 95% CI)^*^		***P*-value**	Density DII category (OR, 95% CI)^**^		***P*-value**	Continuous normal DII (OR, 95% CI)^*^	***P*-value**	Continuous density DII (OR, 95% CI)^**^	***P*-value**
	**DII > -0.12**	**DII ≥ -0.12**		**DII > 0.4**	**DII ≥ 0.4**					
Model A	1 (ref.)	1.6 (1.1–2.3)	0.019	1 (ref.)	2.5 (1.6–3.9)	< 0.001	1.0 (0.8–1.1)	0.993	1.2 (1.1–1.4)	0.015
Model B	1 (ref.)	1.7 (1.1–2.6)	0.007	1 (ref.)	2.8 (1.8–4.4)	< 0.001	0.9 (0.8–1.1)	0.856	1.2 (1.1–1.5)	0.021
Model C	1 (ref.)	1.8 (1.2–2.7)	0.006	1 (ref.)	2.8 (1.8–4.4)	< 0.001	0.9 (0.8–1.2)	0.694	1.2 (1.1–1.5)	0.033

^*^Normal DII, not adjusted DII

^**^Density DII, DII adjusted for energy

Model A, crude ORs; Model B, age and BMI adjusted; Model C, further adjustments for physical activity, mothers’ marital status and educational level and number of siblings; *OR* Odds ratio, *CI* Confidence intervals, DII Dietary inflammatory index

## Discussion

Results of the present study showed that the participants with a lower DII had higher intakes of total fibers, beta-carotene and lutein and lower intakes of total fat, SFA, MUFA, PUFA, oleic acid and linoleic acid, compared with the participants with a higher DII. In addition, the higher DII was associated to higher body fat. Similar to these results, studies reported that higher DII scores were associated to lower healthy food intakes [24]. Andrade et al. followed up 132 obese women for six months after bariatric surgery and reported that women with a higher DII experienced smaller weight and fat mass losses [25]. Associations between dietary components and inflammation have frequently been reported. Western diets, which are rich in SFAs and total fats and low in fruits and vegetables with high DII scores, were reported to increase serum inflammatory markers, including IL-6, homocysteine, and C-reactive protein [2, 26]. However, nutrients such as omega-3, fibers, vitamin E, vitamin C, and β-carotene are associated with lower serum levels of the inflammatory markers [2, 3]. A recent review study has reported that Mediterranean diets, which are rich in fish, fruits, vegetables and, olive oil, may decrease inflammatory factors such as CRP, IL-6, and fibrinogen as well as risks of obesity [27].

In terms of the associations between the DII and obesity, Ruiz-Canela et al. showed that DII included significant associations with abdominal obesity; similar to that of the present study [5]. Aslani et al. reported that pro-inflammatory diets were linked to higher levels of obesity indices such as hip circumference, waist circumference, neck circumference, and abdominal obesity in young children and adolescents [28]. Regarding associations between the DII and body composition, Correa-Rodriguez et al. reported that DII was significantly associated with fat-free mass and weight but not with BMI and fat mass in adults [8]. These contradictory results might be due to the participants’ age and DII might play significant roles in obesity and BF%, majorly in childhood and adolescence. Furthermore, results of a study by Kendal et al. on 81 participants demonstrated that anti-inflammatory diets could be effective in obesity management and significant decreases in body weight and visceral adipose were identified in the intervention group receiving anti-inflammatory diets [7]. Ferreira et al. reported that the use of anti-inflammatory diets was an effective strategy in weight decreases and improvement of obesity-linked comorbidities in obese adolescents [25].

It is possible that associations between body fat and inflammation are mutual. Pre-inflammatory diets may increase body adipose tissues in individuals and higher body fats may increase inflammatory factors [5, 10]. The underlying mechanisms of the effects of inflammation on BMI are not clear. However, a potential mechanism of the higher effects of inflammatory components of diets on the levels of inflammatory factors in obese people includes activation of pathogen-associated molecular pathways such as toll-like and nod-like receptors after receiving pre-inflammatory diets, which induces the production of inflammatory markers in several tissues such as adipose tissues [13, 16]. The DII was reported to be associated with overweight and obesity; however, the effects of inflammatory diets in healthy adults may be negligible [29]. Underlying mechanisms; by which, DII affects BF% are also unclear; however, studies have suggested that pro-inflammatory cytokines such as IL-6, IL-1, and TNF-α can increase appetite and calorie intake in obese people [19]. Furthermore, studies have shown that diets with high DII scores increase CRP levels and thus increase risks of metabolic syndrome [30]. In people with metabolic syndrome, serum levels of circulating adipokines such as omentin-1 and chemerin are altered [31], which include essential roles in the development of obesity and regulation of adipogenesis [32, 33]. However, the results of the present study were limited due to its cross-sectional design and the recall bias due to the nature of the FFQ self-report. Further studies with interventional design are needed to assess associations between dietary-induced inflammations and obesity indices.

## Conclusion

Results of the present study showed that lower scores of DII were associated to lower BF%. Thus, diets low in inflammatory factors might effectively improve body composition and prevent obesity in adolescents. Future comprehensive studies are necessary to verify these findings and to identify the underlying mechanisms of the associations between dietary-induced inflammation and body composition.

## Data Availability

Datasets used and/or analyzed during the current study are available from the corresponding author on reasonable requests.

## Abbreviations

CRP: C-reactive protein
TNF-α: Tumor necrosis factor α
IL-6: Interleukin 6
BMI: Body mass index
DII: Dietary inflammatory index
BF: Body fat percentage
BIA: Bio-impedance analyzer
FFQ: Food frequency questionnaire
